# Group B Streptococcal β-Hemolysin/Cytolysin Directly Impairs Cardiomyocyte Viability and Function

**DOI:** 10.1371/journal.pone.0002446

**Published:** 2008-06-18

**Authors:** Mary E. Hensler, Shigeki Miyamoto, Victor Nizet

**Affiliations:** 1 Department of Pediatrics, University of California San Diego, La Jolla, California, United States of America; 2 Department of Pharmacology, School of Medicine, University of California San Diego, La Jolla, California, United States of America; 3 Skaggs School of Pharmacy & Pharmaceutical Sciences, University of California San Diego, La Jolla, California, United States of America; University of Birmingham, United Kingdom

## Abstract

**Background:**

Group B *Streptococcus* (GBS) is a leading cause of neonatal sepsis where myocardial dysfunction is an important contributor to poor outcome. Here we study the effects of the GBS pore-forming β-hemolysin/cytolysin (Bh/c) exotoxin on cardiomyocyte viability, contractility, and calcium transients.

**Methodology/Principal Findings:**

HL-1 cardiomyocytes exposed to intact wild-type (WT) or isogenic Δβh/c mutant GBS, or to cell-free extracts from either strain, were assessed for viability by trypan blue exclusion and for apoptosis by TUNEL staining. Functionality of exposed cardiomyocytes was analyzed by visual quantitation of the rate and extent of contractility. Mitochondrial membrane polarization was measured in TMRE-loaded cells exposed to GBS βh/c. Effects of GBS βh/c on calcium transients were studied in fura-2AM-loaded primary rat ventricular cardiomyocytes. Exposure of HL-1 cardiomyocytes to either WT GBS or βh/c extracts significantly reduced both rate and extent of contractility and later induced necrotic and apoptotic cell death. No effects on cardiomyocyte viability or function were observed after treatment with Δβh/c mutant bacteria or extracts. The βh/c toxin was associated with complete and rapid loss of detectable calcium transients in primary neonatal rat ventricular cardiomyocytes and induced a loss of mitochondrial membrane polarization. These effects on viability and function were abrogated by the βh/c inhibitor, dipalmitoyl phosphatidylcholine (DPPC).

**Conclusions/Significance:**

Our data show a rapid loss of cardiomyocyte viability and function induced by GBS βh/c, and these deleterious effects are inhibited by DPPC, a normal constituent of human pulmonary surfactant.. These findings have clinical implications for the cardiac dysfunction observed in neonatal GBS infections.

## Introduction

The Gram-positive pathogen Group B *Streptococcus* (GBS, *Streptococcus agalactiae*) is a major cause of pneumonia, sepsis and meningitis in human newborns [1], [2]. Despite recent advances in universal screening and intrapartum antibiotic prophylaxis, GBS remains an important cause of neonatal morbidity and mortality [3]–[5]. In the more common early-onset form of GBS infection, the pathogen gains access to the fetus by ascending infection of the chorioamnion, or alternatively, following aspiration of contaminated vaginal fluids during passage through the birth canal [6], [7]. Severe early onset GBS disease is characterized by systemic hypotension, decreased cardiac output, and arterial hypoxemia, ultimately leading to multiorgan failure [8], [9]. These prominent cardiovascular manifestations are recapitulated experimentally in rabbits, pigs or sheep challenged with GBS by intravenous infusion [10]–[13].

The molecular effectors of GBS-induced cardiac dysfunction have not been determined. GBS cell wall preparations and culture supernatants can activate macrophage/monocytes through multiple Toll-like receptors, leading to the release of abundant cytokines and other proinflammatory mediators that contribute to cell and organ dysfunction during sepsis [6], [14]–[16]. We hypothesized that specific GBS microbial products could also have a direct adverse effect on cardiomyocyte function. Such a phenomenon has recently been postulated for sepsis secondary to *Staphylococcus aureus* and *Escherichia coli*, since exogenous administration of pore-forming bacterial exotoxins produced by each species causes myocardial depression and loss of myocardial contractility in isolated perfused rat hearts [17]–[19].

GBS produces a pore-forming exotoxin known as the β-hemolysin/cytolysin (βh/c) that is responsible for the characterstic zone of clearing (“β-hemolysis”) surrounding colonies grown on blood agar media. Although to date this toxin has not been purified to homogeneity, the discovery of the *cyl* operon encoding the GBS βh/c activity has allowed generation of isogenic βh/c-deficient mutant strains for analysis of toxin function using *in vitro* and *in vivo* models of disease pathogenesis [20], [21]. Within this operon, the *cylE* gene represents a candidate structural gene for the βh/c toxin since heterologous expression of *cylE* confers hemolysis to *E. coli* [21]; however, evidence exists that other GBS *cyl* genes contribute to maximal hemolysin production by the pathogen [20], [22]. GBS βh/c has been shown to contribute to injury of pneumocytes [23], brain endothelial cells [24], and macrophages [25], and to promote GBS-induced mortality in murine or rabbit models of pneumonia [26], arthritis [27], sepsis [28] and meningitis [29]; the effects of this potent bacterial toxin on cardiac function, however, have not been examined.

In the current study, we observe that GBS has the capacity to impair the function and viability of cultured cardiomyocytes. Using wild-type (WT) and isogenic β-h/c-deficient (*cylE* knockout) mutant GBS, together with cell free extracts from these bacteria, we determine the β-h/c toxin to play a principle role in this cardiotoxicity. Inhibition of β-h/c activity abrogates these changes, suggesting toxin neutralization could represent a pharmacological target to reduce cardiac complications in GBS neonatal sepsis.

## Results

### GBS βh/c impairs cardiomyocyte contractility

**Figure 1:**
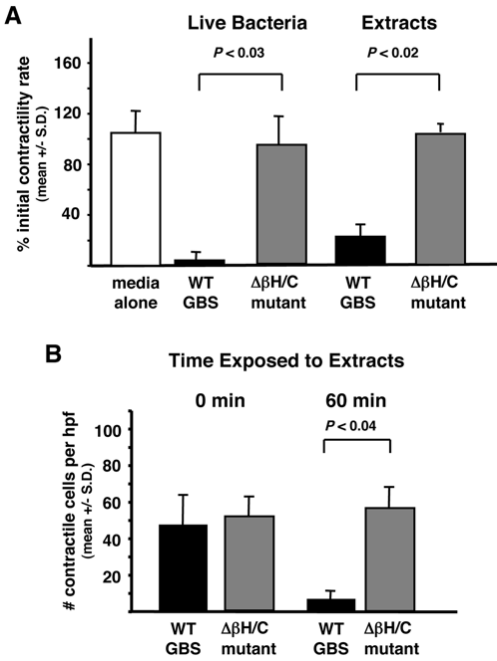
GBS βh/c effects on HL-1 cardiomyocyte contractility. (A) Contractility rate (beats per min) was determined for HL-1 cells before and 1 hr after exposure to live WT or Δβh/c mutant GBS bacteria (multiplicity of infection = 1.0) or hemolysin extracts (1∶100). Graphs depict percentage of the initial contractility rate following treatment. (B) Number of contractile cells per high power field (hpf) prior to and 60 min after exposure to extracts from WT or Δβh/c mutant GBS. Data represent the mean +/− SD from three separate experiments.

HL-1 cardiomyocytes become synchronously contractile in culture, and this contractility can be easily quantitated by determining the rate (in beats per minute) and extent (number of contractile cells per high power field) of contractile activity. To determine the effect of GBS and its βh/c toxin on cardiomyocyte function, contractile HL-1 cardiomyocytes were incubated with either WT GBS or Δβh/c GBS and contractility rate assessed. Exposure of HL-1 cells to WT GBS at an MOI = 1.0 produced a pronounced decrease in the rate of contractile activity within 1 h compared to cells treated with an equivalent number of Δβh/c GBS (Fig. 1A). WT GBS exposed cardiomyocytes that remained contractile after 1 h exhibited marked asynchronous contractile activity. Similar effects of WT but not Δβh/c mutant GBS on cardiomyocyte contractility were also noted at an MOI = 10 (data not shown). When cell-free extracts of GBS were prepared and added to the cardiomyocytes at 1∶100 dilution, only the extracts from WT and not Δβh/c mutant bacteria had a significant effect on rate and extent (number of contractile cells per high power field) of HL-1 contractility at 1 h (Fig. 1A and B). An approximate 60% reduction in cardiomyocyte function was observed at a WT extract dilution as high as 1∶10,000 and as early as 30 min after exposure (data not shown). No change in contractile activity was noted for cells exposed to the extract prepared from Δβh/c GBS even following 4 h exposure (data not shown). These results suggest that GBS βh/c exerts a direct and rapid effect on cardiomyocyte function.

### GBS βh/c impairs cardiomyocyte viability

**Figure 2:**
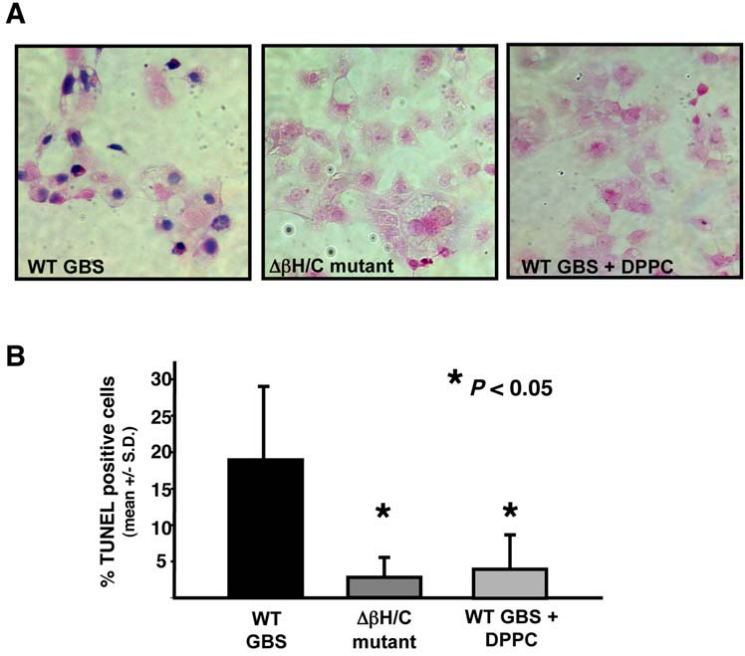
GBS βh/c effects on HL-1 cardiomyocyte viability. (A) Trypan blue staining of HL-1 cells exposed for 1 h to extracts (1∶200 dilution) from WT GBS in the absence or presence of DPPC (2 mg/ml) or to similar extracts of Δβh/c mutant GBS. (B) Analysis of TUNEL staining by flow cytometry. Percent TUNEL positive HL-1 cells that had been treated for 1 h with extracts (1∶200) of WT GBS in the absence or presence of 2 mg/ml DPPC or with extracts from Δβh/c mutant GBS. Apoptosis data represent the mean +/− SD from three separate experiments.

To determine if GBS βh/c was inducing cardiomyocyte cell death, both trypan blue staining and TUNEL staining for apoptosis were performed on cells exposed to cell free GBS extracts. Trypan blue staining of HL-1 cells after 1 h of exposure to extract from WT GBS showed high percentages of dead cells (Fig. 2A), while such changes were absent in cells treated with extract of the Δβh/c mutant. DPPC is the major component of pulmonary surfactant, and its relative absence in the lungs of premature neonates is believed to contribute to a worse prognosis for GBS-infected premature infants [30], [31]. Addition of DPPC (2 mg/ml), a known inhibitor of βh/c toxicity to erythrocytes and epithelial cells [23], [32], blocked the cell injury induced by WT GBS extract (Fig. 2A). Because GBS βh/c has been shown to induce apoptosis in mammalian cells [25], we investigated whether evidence of apoptosis could be detected at early timepoints in hemolysin-exposed cardiomyocytes. After 1 h, a significantly greater percentage of HL-1 cardiomyocytes exposed to WT GBS extract were positive for TUNEL stain compared to those exposed to Δβh/c mutant extract; and the degree of TUNEL staining was significantly reduced in the presence of DPPC (Fig 2B). Pretreatment of HL-1 cells with either EGTA (1 mM) to chelate extracellular calcium or with the cell-permeable calcium chelator, BAPTA-AM (1 nM), each sufficient to inhibit contractile activity, had no effect on cell death analyzed by either trypan blue or TUNEL staining (data not shown), suggesting that the induction of cell death did not depend on significant levels of intracellular calcium. Collectively, these data suggest that βh/c induces a rapid loss of cardiomyocyte function and leads to cell death, possibly involving both necrotic and apoptotic pathways.

### Effects of βh/c on cardiomyocyte calcium transients

**Figure 3:**
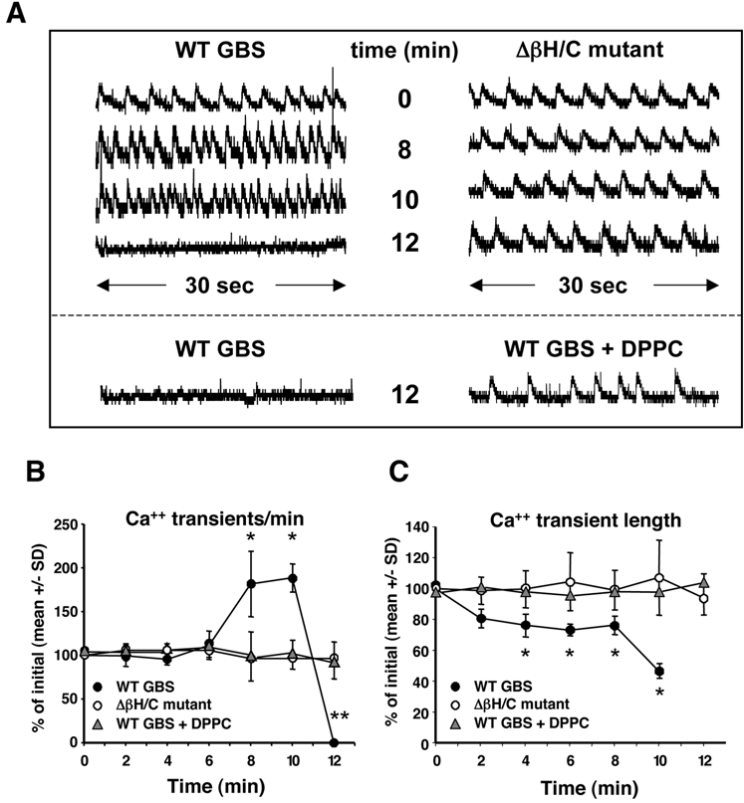
Calcium transients of primary rat neonatal ventricular cardiomyocytes upon exposure to GBS βh/c (1∶200 dilution). (A) Representative 30 sec calcium transient profiles of primary neonatal ventricular cardiomyocytes exposed to extracts of WT or Δβh/c mutant GBS or to WT extracts in the presence of 2 mg/ml DPPC for up to 12 min. (B) The number of calcium transients per minute was calculated prior to and during exposure to extracts of either WT or βh/c mutant GBS. Data represent the percent of initial (at time 0) number of calcium transients per minute +/− SD from three independent experiments; *P<0.04, **P<0.03. (C) Length of calcium transients was calculated from five randomly selected transients for each 30 sec recording. Data represent the percent of initial (at time 0) average length of calcium transient mean +/− SD from three independent experiments; *P< 0.05.

Because the GBS βh/c toxin has previously been shown to form pores in target cell membranes with the potential for disrupting cytosolic ion concentrations, cytosolic calcium levels during exposure to hemolysin were assessed using the ratiometric dye, fura-2. Fura-2AM-loaded neonatal rat primary cardiomyocytes were treated with either GBS WT or Δβh/c mutant extracts, and calcium transients observed at 2 min intervals over time. Normal-appearing calcium transients were observed in the first several min following exposure to either WT or Δβh/c mutant extracts. However, by 8 min, the calcium transient rate (number of transients per minute) markedly increased for cells exposed to WT but not Δβh/c mutant GBS extract (Fig. 3A and B). Furthermore, by 10 min, the length of calcium transients markedly decreased in primary cardiomyocytes exposed to the WT extract, with a slight and statistically significant decrease observed starting at 4 min (Fig. 3C). Baseline cytosolic calcium levels also appeared slightly elevated starting at 12 min of exposure to GBS WT Extract and remained so out to at least 20 min (data not shown), perhaps suggesting an unregulated entry of extracellular calcium into cells, becoming apparent when calcium transients were no longer detectable. Both the rate and length of transients remained unchanged at all timepoints studied up to 20 min for primary cardiomyocytes exposed to extracts from the GBS Δβh/c mutant (Fig. 3A–C) or with buffer alone (data not shown). The detrimental effect of GBS βh/c on calcium transients was fully inhibited by co-treatment with 2 mg/ml DPPC (Fig. 3A–C).

### GBS βh/c disrupts mitochondrial membrane potential in cardiomyocytes

**Figure 4:**
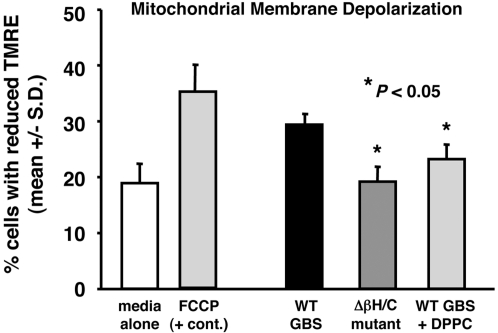
TMRE staining of HL-1 cells exposed to βh/c extracts. HL-1 cardiomyocytes were loaded with TMRE (50 nM) for 30 min prior to exposure to WT or Δβh/c mutant hemolysin extracts (1∶200 dilution) or FCCP (5 uM) for 1 h. Cells were rinsed in DPBS, trypsinized, and analyzed for TMRE staining by flow cytometry. Data represent mean percent of cells with reduced TMRE staining +/− SD from three separate experiments.

Because of its importance in cell viability and function, the effect of βh/c on mitochondrial membrane potential was investigated. HL-1 cells were loaded with the mitochondrial membrane potential indicator, TMRE, and loaded cells were treated with extracts of WT or Δβh/c mutant GBS for 1 h. Membrane potential was assessed by FACS analysis of TMRE staining of trypsinized cells. As shown in Fig. 4, cells exposed to extracts of WT GBS exhibited a high percentage of cells with reduced TMRE staining, indicative of a loss of mitochondrial membrane potential, approaching the loss of potential observed after treatment with the positive control agent, FCCP. Conversely, the percentage of GBS Δβh/c extract-exposed cardiomyocytes with reduced TMRE staining did not differ significantly from media control (Fig. 4). Finally, the effect of WT GBS βh/c extract on TMRE staining was significantly reduced when cells were co-treated with DPPC (Fig. 4).

## Discussion

The precise etiology of mortality in early-onset GBS sepsis is not known, but development of hypotension is a discriminating feature and an important predictor of fatal outcome [8], [9]. Experiments in a rabbit model following intravenous infusion of GBS revealed a dramatic decrease in the first derivative of left ventricular pressure over time (LVdP/dt), decreased cardiac output, and decreased mean arterial pressure, yet no changes in systemic vascular resistance or left ventricular end diastolic pressure, a pattern that points to a primary role for myocardial dysfunction in GBS sepsis [11]. Our present study examined for the first time the direct interaction between this pathogen and cardiomyocytes, using an *in vitro* tissue culture system. We find that GBS induces cardiomyocyte dysfunction and promotes cardiomyocyte death in a fashion that depends upon expression of its β-h/c toxin.

Previous studies of the role of GBS β-h/c in murine or rabbit models of invasive infection have not examined cardiac function directly, although the presence of the toxin was significantly correlated to development of hypotension [28], [33] and overall mortality [25], [27], [29]. Here we show a direct effect of GBS βh/c on cardiomyocyte viability and contractility as well as in maintenance of normal calcium transients. We observed that in cardiomyocytes exposed to GBS βh/c, the rate of calcium transients per minute was greatly increased, and the average length of transients was significantly decreased, immediately prior to complete elimination of transient formation. Previous studies have shown that early apoptotic changes in adult cardiomyocytes include unusual contractile activity and increases in calcium transients that are believed to be involved in the initiation of the cell death pathway [34]. The rapid pore-forming activity of GBS βh/c appears to accelerate the kinetics of processes such as increases in calcium transient frequency in comparison to other pathologic conditions such as chronic cardiac failure. It is possible that parallel pathophysiologic changes are induced by other bacterial pore-forming toxins such as *S. aureus* α-toxin and *E. coli* hemolysin, which have been shown to affect contractility of isolated perfused hearts [17], [18], but have yet to be studied in isolated cardiomyocytes for potential effects on calcium transients or apoptosis.

Our data show a rapid loss of cardiomyocyte contractility and viability induced by GBS through the action of its βh/c toxin, which may involve both necrotic and apoptotic mechanisms. We observed effects on cardiomyocyte functionality within minutes of exposure to the toxin. A detectable rise in intracellular calcium within 20 min of βh/c exposure may precipitate downstream effects such as loss of mitochondrial membrane polarization, as apparent in our TMRE studies after 1 h. Recently, inhibition of mitochondrial membrane permeability transition using immunosuppressive agents or mitochondrial Bcl-2 overexpression has been shown to inhibit sepsis-induced myocardial dysfunction and mortality in a mouse model [35]. Our finding that βh/c-induced apoptosis was not inhibited by intracellular and extracellular calcium chelation suggests this particular process could be calcium-independent.

We hypothesize that βh/c secreted and accumulated during systemic neonatal infection with GBS could affect maintenance of normal calcium transients in intact cardiomyocytes and potentially lead to cell death in localized areas of cardiac tissue, thereby promoting asynchronous contractile activity and myocardial dysfunction and even death. In our previous work examining GBS pulmonary infection in neonatal rabbits, developmentsystemic infection was correlated to β-h/c activity [26], and we report here an example of a GBS microabscesses within the cardiac tissue of a rabbit pup infected with the WT strain from that study (Figure S1). GBS is also an uncommon yet well-documented cause of infective endocarditis in adult populations. GBS endocarditis is an aggressive disease with a high rate of local and systemic complications, with ∼40% of patients requiring surgery because of extensive valve destruction and fatal outcome in more than 1/3 of patients [36], [37]. It is possible therefore that GBS βh/c induced cardiomyocyte cell death also contributes to the poor prognosis of this syndrome.

Our earlier published work has demonstrated a definitive role for βh/c in cytotoxicity to lung epithelial cells [23], pulmonary endothelial cells [38], brain endothelial cells [24], and macrophages [25]. Together with the findings of the present study regarding cardiomyocytes, this broad-spectrum cytolysin likely contributes in multiple fashions to the life-threatening manifestations of severe early onset GBS disease. Pharmacological agents, including phosopholipid DPPC, that are designed to block βh/c cytotoxicity could represent a therapeutic strategy to mitigate cardiac and other organ dysfunction during such infections. Of note, DPPC is already used as a major component of surfactant administered intratracheally in replacement therapy for respiratory distress syndrome of the neonate and certain liposomal preparations for delivery of systemic agents (e.g. amphotericin).

## Methods

### Cells and Reagents

HL-1 atrial cardiomyocytes [39] were a kind gift of Dr. William Claycomb (Louisiana State University Medical Center). Neonatal rat ventricular cardiomyocytes were isolated from rat pups and as previously described [40]. Our investigations conform with the *Guide for the Care and Use of Laboratory Animals* published by the US National Institutes of Health (NIH Publication No. 85-23, revised 1996). Todd-Hewitt broth (THB) and agar (THA) were obtained from Hardy Diagnostics (Santa Maria, CA). GBS strains used were NCTC 10/84 (hereafter designated WT), a hemolytic serotype V clinical isolate from neonatal sepsis [23], and its corresponding isogenic β/hc-deficient allelic replacement mutant NCTC:*cyl*EΔ*cat* [21], hereafter designated Δβh/c. Fura-2AM, APO-Brdu TUNEL Assay Kit, BAPTA-AM, FCCP (carbonylcyanide p-(trifluoromethoxy)phenylhydrazone), and TMRE (tetramethylrhodamine ethyl ester) were each obtained from Invitrogen/Molecular Probes (Eugene, OR). Fibronectin used to coat tissue culture dishes and plates and the βh/c inhibitor DPPC (1,2 dipalmitoyl-sn-glycero-3-phosphocholine) were obtained from Sigma-Aldrich (St. Louis, MO).

### GBS βh/c and DPPC preparation

Partially purified hemolysin extracts were prepared using a modification of a previously published protocol [32]. Briefly, overnight cultures of WT and Δβh/c GBS grown in parallel in THB were diluted 1∶20 in 500 ml fresh THB and grown to A_600_ = 0.4. The bacteria were then pelleted and washed twice in Dulbecco's phosphate-buffered saline (DPBS - Gibco). Washed bacteria were next resuspended in DPBS containing 1% glucose and 1% soluble starch (Difco) and incubated for 1 h at 37°C. Bacteria were then pelleted, and supernatant containing starch-stabilized βh/c was 0.2 µm-filtered and then precipitated in an equal volume of ice-cold methanol. The methanol precipitate was dried, resuspended in 1 ml of PBS, filter-sterilized, and stored at 4°C. Each WT and Δβh/c mutant GBS extract used in these experiments was assayed for hemolytic titer on sheep erythrocytes prior to use in cardiomyocyte experiments; extracts from WT GBS demonstrated hemolytic activity following dilution to 1∶2500, while Δβh/c mutant extracts exhibited no hemolytic activity at a 1∶20 dilution. DPPC was prepared fresh for each experiment by sonicating 20 mg/ml DPPC in media on a narrow tip sonicator (550 Sonic Dismembrator, Fisher Scientific, Pittsburgh, PA) for two 45 second pulses (setting 3).

### Cardiomyocyte viability and contractility assays

HL-1 cardiomyocytes were grown in 5% CO_2_ at 37°C in supplemented Claycomb media as previously described [41]. For assays of viability and contractility, ∼10^5^ cells/well were plated in fibronectin-coated 12 well plates (Becton-Dickinson, Franklin Lakes, NJ) and allowed to grow for 2–3 days until contractile activity was observed. On the day of the assay, media was replaced with fresh media, and cells were allowed to equilibrate for an additional 1 h at 37°C. For assays using intact bacteria, WT and Δβh/c mutant GBS were grown to A_600_ = 0.4, washed twice in DPBS, and resuspended to A_600_ = 0.4 in Claycomb media. Washed bacteria were added at a multiplicity of infection (MOI) = 1.0 and incubated with the cells for 1 h prior to assessment of cardiomyocyte viability or contractility. WT and Δβh/c cell-free extracts were added to the HL-1 cells at known dilutions ranging from 1∶100 to 1∶10,000 and incubated as for the intact bacteria. Assessment of cell viability and contractility was performed using a Zeiss Axiovert 40 CFL inverted microscope. For viability studies, cells were gently rinsed three times in DPBS and fixed in 1% formalin for 30 min prior to trypan blue and eosin staining. Contractility rate was enumerated from a 30 sec count of the synchronously contractile cells in three random fields. Number of contractile cells per high power field (hpf) at 400× magnification was assessed for three randomly-chosen fields.

### Apoptosis assays

HL-1 cardiomyocytes were added to 6-well plates (BD Falcon, Franklin Lakes, NJ) at ∼5×10^5^ cells per well and incubated for 2 d prior to assay. For the assay, media was replaced with fresh media, and hemolysin extracts prepared from WT or Δβh/c mutant bacteria added at 1∶200 dilution. Where indicated, freshly-prepared DPPC was added at a final concentration of 2 mg/ml at the same time as the hemolysin. After 1 h, the cells were gently rinsed in DPBS, trypsinized, and fixed in 1% formalin for 30 min. Cells were end-labeled per Apo-Brdu TUNEL Assay kit instructions and then analyzed for staining by flow cytometry (BD FACSCalibur).

### Measurement of calcium transients

Freshly-isolated rat cardiomyocytes were plated at 0.9×10^6^ cells on fibronectin-coated 35 mm glass bottomed dishes and allowed to grow for 2 d prior to use. Media was replaced daily until assay, and cells were assessed for contractile activity prior to fura-2AM treatment. Cells were loaded with 5 µM fura-2AM at room temperature for 30 min in clear DMEM followed by two rinses in clear DMEM and a 30 min incubation to allow full cleavage of fura-2AM to fura-2. Cells were then analyzed on an inverted microscope, and fluorescence recorded with a dual excitation fluorescence photomultiplier system (IonOptix) as previously described [42]. Baseline recording was performed prior to addition of hemolysin extracts (1∶200 dilution), and calcium transients were recorded for 30 sec time intervals every 2 min for up to 20 min. Where indicated, DPPC was added at 2 mg/ml final concentration at the time of hemolysin addition. Both the number of calcium transients per 30 sec and the length of the transients at baseline (from an average of five randomly-chosen transients per 30 second recording) were calculated for each group. Data reported represent the average of three separate experiments.

### Mitochondrial membrane depolarization studies

HL-1 cardiomyocytes were plated as for the apoptosis studies in section 2.4. Cells were loaded with 50 nM TMRE for 30 min at 37°C and rinsed gently prior to addition of hemolysin preparations (1∶200) for one hour. Cells were then rinsed in DPBS, trypsinized, and analyzed for TMRE staining by flow cytometry (BD FACSCalibur) at 582 nm. As a positive control for mitochondrial membrane depolarization, cells were exposed to the H^+^ ionophore, FCCP (5 µM), for 30 minutes prior to analysis.

### Statistical analysis

Data are expressed as mean +/− SD. Between group differences were analyzed by Mann-Whitney U-test. Differences were considered statistically significant at *P*<0.05.

## Supporting Information

Figure S1 Micrograph of group B Streptococcus microabscess in cardiac tissue of neonatal rabbit infected by direct intratracheal inoculation with resultant bacteremia (challenge model described in Hensler et al. J Infect Dis 2005; 191:1287-91). Cell death and necrosis of surrounding cardiomyocytes is apparent.
(7.34 MB TIF)
