# Supplementary figures and images for: Group B Streptococcal β-Hemolysin/Cytolysin Directly Impairs Cardiomyocyte Viability and Function
Source: PLoS One. 2008 Jun 18;3(6):e2446. doi: 10.1371/journal.pone.0002446 (PMC2409074; doi:10.1371/journal.pone.0002446)

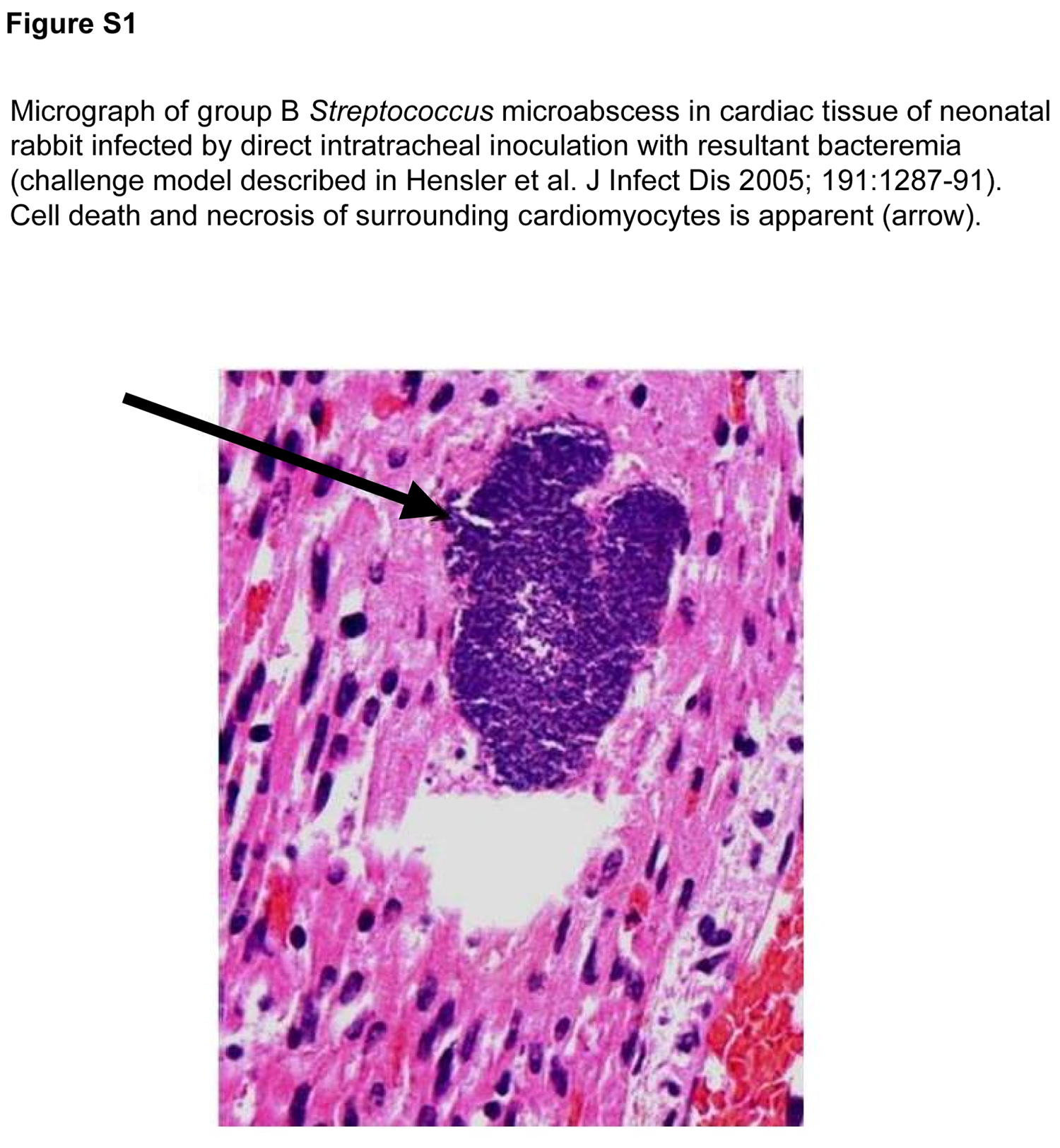

Supplement: Figure S1 — Micrograph of group B Streptococcus microabscess in cardiac tissue of neonatal rabbit infected by direct intratracheal inoculation with resultant bacteremia (challenge model described in Hensler et al. J Infect Dis 2005; 191:1287-91). Cell death and necrosis of surrounding cardiomyocytes is apparent. (7.34 MB TIF) [file pone.0002446.s001.tif]
